# Identification of small-molecule allosteric modulators that act as enhancers/disrupters of rhodopsin oligomerization

**DOI:** 10.1016/j.jbc.2021.101401

**Published:** 2021-11-11

**Authors:** Tamar Getter, Albert Kemp, Frans Vinberg, Krzysztof Palczewski

**Affiliations:** 1 Department of Ophthalmology, Gavin Herbert Eye Institute, University of California, Irvine, California, USA; 2 Department of Ophthalmology and Visual Sciences, University of Utah, Salt Lake City, Utah, USA; 3 Department of Physiology and Biophysics, University of California, Irvine, California, USA; 4 Department of Chemistry, University of California, Irvine, California, USA; 5 Department of Molecular Biology and Biochemistry, University of California, Irvine, California, USA

**Keywords:** rhodopsin, dimerization, G protein–coupled receptor, phototransduction, high-throughput screening, rod photoreceptors, retina, β-gal, β-galactosidase, BRET, bioluminescence resonance energy transfer, DMSO, dimethyl sulfoxide, EA, large β-gal subunit, ERG, electroretinogram, GPCR, G protein–coupled receptor, HTS, high-throughput screening, LMNG, lauryl maltose-neopentyl glycol, Meta, metarhodopsin, PK, small subunit of β-gal, *R*_max_, maximal response amplitude, ROS, rod outer segment, S/B, signal-to-background, U2OS, human osteosarcoma

## Abstract

The elongated cilia of the outer segment of rod and cone photoreceptor cells can contain concentrations of visual pigments of up to 5 mM. The rod visual pigments, G protein–coupled receptors called rhodopsins, have a propensity to self-aggregate, a property conserved among many G protein–coupled receptors. However, the effect of rhodopsin oligomerization on G protein signaling in native cells is less clear. Here, we address this gap in knowledge by studying rod phototransduction. As the rod outer segment is known to adjust its size proportionally to overexpression or reduction of rhodopsin expression, genetic perturbation of rhodopsin cannot be used to resolve this question. Therefore, we turned to high-throughput screening of a diverse library of 50,000 small molecules and used a novel assay for the detection of rhodopsin dimerization. This screen identified nine small molecules that either disrupted or enhanced rhodopsin dimer contacts *in vitro*. In a subsequent cell-free binding study, we found that all nine compounds decreased intrinsic fluorescence without affecting the overall UV-visible spectrum of rhodopsin, supporting their actions as allosteric modulators. Furthermore, *ex vivo* electrophysiological recordings revealed that a disruptive, hit compound #7 significantly slowed down the light response kinetics of intact rods, whereas compound #1, an enhancing hit candidate, did not substantially affect the photoresponse kinetics but did cause a significant reduction in light sensitivity. This study provides a monitoring tool for future investigation of the rhodopsin signaling cascade and reports the discovery of new allosteric modulators of rhodopsin dimerization that can also alter rod photoreceptor physiology.

Rod photoreceptor cells are highly differentiated neurons composed of four anatomically distant regions: the rod outer segment (ROS), inner segment, nucleus, and synaptic termini (1, 2). In mice, the ROS is composed of about 600 internal flattened discs surrounded by plasma membranes (3). Rhodopsin is an integral component of the disc and plasma membranes. Expression of rhodopsin is tightly regulated, and genetic ablation of two copies of the gene prevents formation of the ROS beyond rudimentary structures (4, 5). Deletion of one copy of the rhodopsin gene led to reduction of the volume of the ROS to approximately 60% in rhodopsin^+/−^ mice (3). Overexpression of rhodopsin in rod photoreceptors increased their ROS diameters, providing additional membranes to accommodate a larger number of rhodopsin molecules (6). In both cases, when rhodopsin expression was reduced or overexpressed, the overall rhodopsin packing density did not change (3, 6). The ROS has the highest density of any G protein–coupled receptor (GPCR) in nature, equating to about 5 mM concentration. Taking the size, molecular mass, and ratio of lipids per rhodopsin into account, it was estimated that the average ratio would be approximately 54 to 86 phospholipids/rhodopsin (3). These values are in agreement with atomic force microscopy imaging that estimated an average density between 30,000 and 55,000 rhodopsins per μm^2^ surface area (3, 7, 8, 9). Such density along with the propensity of rhodopsin to oligomerize, a property that is common to all classes of GPCRs (10, 11, 12, 13), leads to formation of oligomeric clusters of the receptor (14). Structural studies further support this view of rhodopsin, as exemplified in several reports (15, 16, 17).

GPCR oligomerization is of importance from three fundamental perspectives: structural, functional, and pharmacological, as briefly discussed here. Despite low homology (18), all GPCRs share convergent structural topology and fold (19, 19, 20, 21). They frequently organize in specific domains of the cell (22, 23, 24). These microdomains organize all elements of the signaling pathway, increasing the dwell time for the ligand and increasing the sensitivity, speed, and selectivity of cellular signaling (14, 25). Thus, GPCR microdomains could be responsible for the precise spatial and temporal control of downstream signaling (14, 26). Homodimerization and heterodimerization could have a profound effect on the function of these GPCRs in terms of ligand or effector selectivity, desensitization, or internalization (27, 28). Finally, this oligomerization potentially offers more selectivity for discovery/development of pharmacological agents besides traditional allosteric or orthosteric ligands because a specific pair of GPCRs could present unique binding sites in a cell- or tissue-specific manner. A focus on drugs affecting dimers of GPCRs offers additional novel opportunities for this most druggable family of receptors (29, 30, 31, 32, 33). Developing small chemical ligands that affect GPCR oligomerization, either enhancers or disrupters, also would improve our understanding of the chemistry and biology of these signaling receptors. Native rod photoreceptor phototransduction is an ideal focus of study due the specialized signaling structures (ROS), precise signaling trigger (light), and phenomenally well-characterized biochemistry and physiology of this system.

Most studies on GPCRs have relied on heterologous expression systems, which represent a good first step, highly amenable to pharmacological and biochemical analyses; however, the data must be considered with caution as to whether they represent physiologically relevant phenomena. Here, we present a hybrid approach, using a high-throughput screen to identify compounds that enhance or disrupt the oligomerization of rhodopsin in heterologous expression systems. Then, the identified lead compounds are tested on native retinas with biochemical and electrophysiological assays for authentication, before further extensive medicinal chemistry is undertaken.

## Results

### HTS identifying rhodopsin dimerization disrupters/enhancers *in vitro*

**Figure 1:**
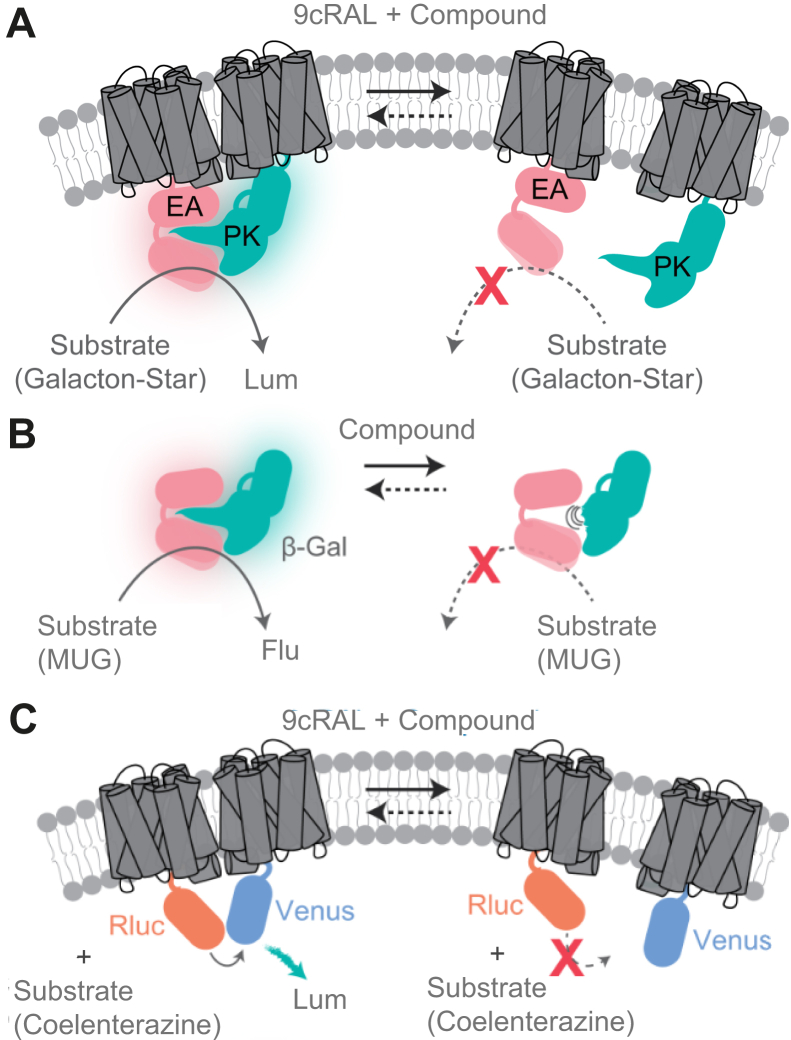
**High-throughput screening (HTS) assay systems.***A*, schematic illustration of the rhodopsin–β-gal fragment HTS assay. Cell membrane is shown expressing opsin-EA and opsin-PK proteins containing the complementary subunits of β-gal (EA [*pink*] and PK [*green*]) fused onto the C-terminal of opsin (*gray*), which are regenerated for phototransduction by 9-*cis*-retinal (9cRAL) after treatment with the screened compounds. Disruption or enhancement of β-gal dimeric substrate reconstitution is quantified by its luminescence signal reduction or elevation, respectively. *B*, schematic illustration of the rhodopsin dimerization BRET complementation assay. Cell membrane is shown expressing opsin-Rluc (*gray* and *orange*) and opsin-Venus (*gray* and *blue*), poised to utilize the FRET reaction *via* luciferase substrate. Cells are regenerated for phototransduction by 9-*cis*-retinal after treatment with screened compounds. Disruption or enhancement of rhodopsin dimerization results in luminescence signal decrease or increase, respectively. *C*, enzymatic activity of β-gal. Schematic representation of β-gal activation and deactivation with hit compounds, followed by substrate treatment generating fluorescence signals as EA (*pink*) and PK (*green*) subunits are complemented. β-gal, β-galactosidase; BRET, bioluminescence resonance energy transfer; EA, large β-gal subunit; Lum, luminescence; MUG, 4-methylumbelliferyl-β-D-galactopyranoside; PK, small subunit of β-gal.

To identify small molecules that target rhodopsin dimerization, a cell-based high-throughput screening (HTS) assay was used to screen a diverse library of 50,000 compounds from Life Chemicals. The human osteosarcoma (U2OS) stable cell line was used, which expresses opsin fused with complimentary subunits of β-galactosidase (β-gal), namely, the enzyme acceptor large β-gal subunit (EA) and the enzyme donor, small subunit of β-gal (PK). Upon rhodopsin self-association *in vitro*, a competent β-gal was formed and chemiluminescent signal was generated in the presence of β-gal substrate (Fig. 1*A*). For further validation, the β-gal activity assay was used to counter-screen and rule out false positives, affecting the activity of β-gal without affecting rhodopsin (Fig. 1*B*) (34). In an orthogonal assay, rhodopsin dimerization was assessed using a bioluminescence resonance energy transfer (BRET) assay with human embryonic kidney 293 cells expressing opsin fused with Renilla luciferase and opsin fused with Venus (Fig. 1*C*) (35, 36). To form functional visual pigment, opsin-fused proteins were regenerated with 9-*cis*-retinal to form so-called isorhodopsin (37). We chose 9-*cis*-retinal over photoreceptor-endogenous 11-*cis*-retinal because of its availability and the prohibitive cost for HTS assays with the native chromophore. Rhodopsin and isorhodopsin have similar biochemical and spectral properties (see (38)).

**Figure 2:**
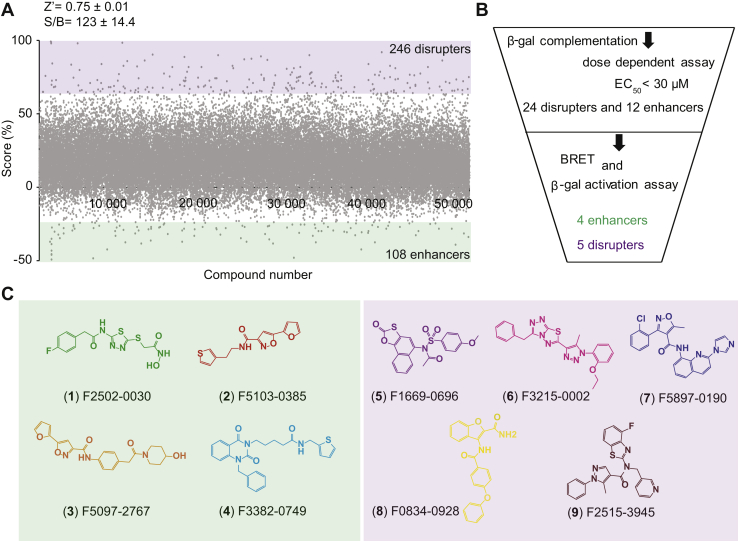
**Rhodopsin dimerization disruption/enhancement HTS.***A*, the rhodopsin–β-gal complementation screen in U2OS cells is illustrated here, showing the activity score plot of 50K screened compounds, where 246 disrupters and 108 enhancer hits were obtained (activity score ≥50%; disrupters: *purple* and enhancers: *green*). Activity scores were normalized by the luminescence measured by opsin-EA–expressing cells *versus* opsin-EA/PK–expressing cells as 0 and 100% controls, respectively. The HTS quality control values S/B ratio and Z′ are shown (*inset*). *B*, hit compound filtering diagram. The rhodopsin–β-gal complementation screen identified 24 disrupters and 12 enhancer hits. The orthogonal validation screen by rhodopsin–BRET and β-gal activity verified the final five disrupter hits (*purple*) and four enhancer hits (*green*). *C*, chemical structure representations of four enhancer (*green*) and five disrupter (*purple*) HTS hits. β-gal, β-galactosidase; EA, large β-gal subunit; HTS, high-throughput screening; PK, small subunit of β-gal; S/B, signal-to-background; U2OS, human osteosarcoma.

All 50,000 compounds were screened, using the β-gal complementation assay at a final concentration of 60 μM with a Z′ factor of 0.75 ± 0.01 and a signal-to-background (S/B) ratio of 123 ± 14.4 (Fig. 2*A*) (39). The activity scores were calculated according to the following equation:

$$\left({\text{RLU}}_{\left(\text{compound}\right)}-{\text{RLU}}_{\left(\text{positive}\;\text{control}\right)}\right)/\left({\text{RLU}}_{\left(\text{negative}\;\text{control}\right)}-{\text{RLU}}_{\left(\text{positive}\;\text{control}\right)}\right)\times 100.$$

The positive control comprised U2OS cells transfected with both opsin-EA and opsin-PK, mimicking dimer formation (with a 0% score value), and the negative control comprised U2OS cells transfected with opsin-EA only, mimicking disrupted dimer formation (with a 100% score value).

Initially, 246 compounds were identified as disrupter molecules of isorhodopsin dimerization, along with 108 compounds that were identified as isorhodopsin dimerization enhancer molecules with negative score values mimicking increases in dimer formation. All identified hits were subjected to triplicate evaluation and dose-dependence studies. In addition, to eliminate the possibility that the decrease in the luminescence signal associated with isorhodopsin dimer disruption was the result of compound cytotoxicity leading to cell death, nuclear morphology of the cells treated with hit compounds was visually inspected by staining the cells with Hoechst 33342, and cell viability was visually confirmed for all tested compounds, as described in prior publications (40, 41).

Identified hits with EC_50_ values lower than 30 μM included 24 disrupting and 12 enhancing molecules. These compounds were subjected to our orthogonal BRET assay for further validation. In this assay, we narrowed our pool of active compounds to five disrupting compounds and four enhancing compounds that were positive in both the β-gal complementation and BRET assays (Fig. 2, *B* and *C*).

**Figure 3:**
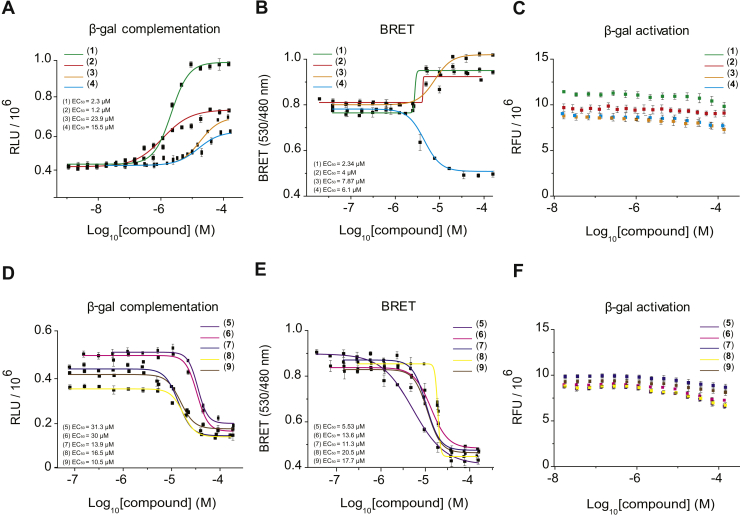
**Dose-dependent effects with rhodopsin dimerization disruption/enhancement hits.***A* and *D*, rhodopsin–β-gal fragment complementation signal increases or decreases *via* treatment with enhancer or disrupter compounds, respectively. Dose–response curves are shown for nine verified rhodopsin–β-gal complementation hits with corresponding EC_50_ values ranging from 30 μM to 1 μM. Error bars correspond to SDs of triplicate readings. *B* and *E*, rhodopsin–BRET assay signal increases or decreases *via* treatment with hit-enhancer or hit-disrupter compounds, respectively. Dose–response curves for nine verified BRET assay hits with corresponding EC_50_ values ranging from 20 μM to 2 μM. Error bars correspond to SDs of triplicate readings. *C* and *F*, enzymatic activity of β-gal *via* treatment with enhancer or disrupter compounds had no dose-dependent effect on β-gal deactivation. Concentrations ranged from 320 μM down to 2.4 nM. Error bars correspond to SDs of triplicates. β-gal, β-galactosidase; BRET, bioluminescence resonance energy transfer.

Next, β-gal complementation (Fig. 3, *A* and *D*) and BRET assays (Fig. 3, *B* and *E*) were used to determine the dose dependence of stimulation or inhibition of isorhodopsin dimerization by these compounds. For all of these active compounds, the EC_50_ values were within the 1 μM to 30 μM range (Fig. 3). Finally, all nine compounds were tested for their β-gal activity to rule out false-positive compounds that affect the activity of this enzyme, but not the rhodopsin dimerization (Fig. 1*B*). In the tested range of concentrations, none of the disrupters or enhancers affected β-gal activity (Fig. 3, *C* and *F*).

An interesting phenomenon was observed for compound #4 (Fig. 3). This compound disrupted dimerization in the β-gal complementation assay, but enhanced dimerization in the BRET assay, suggesting that the orientation of the opsin-fused proteins plays a critical role that may affect dimerization *in vitro*.

### Spectral properties of photoactivated isorhodopsin in the presence of the identified hits

**Figure 4:**
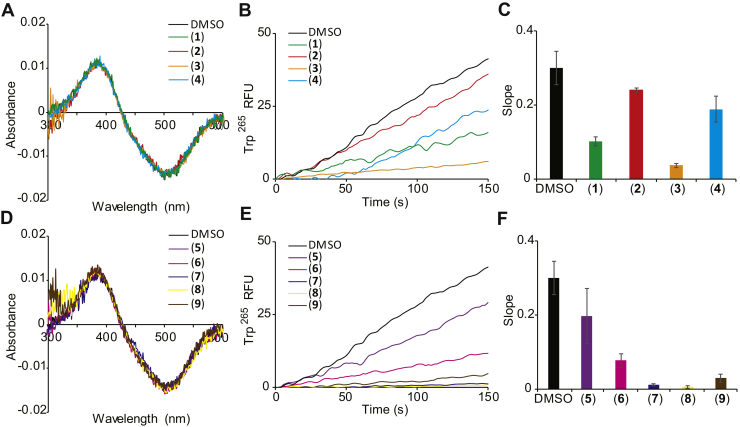
**Effects of enhancers and disrupters on rhodopsin spectral properties.***A* and *D*, UV-absorption spectra of dark and 10-s-irradiated bovine rhodopsin. Calculated differences in spectra were obtained by subtracting the light spectrum from the dark spectrum of rhodopsin preincubated for 10 min at 20 °C with enhancer or disrupter HTS hits, respectively. All nine hit compounds had no effect on rhodopsin UV-absorption compared with the spectra in the DMSO control solution. *B* and *E*, rhodopsin-Trp^265^ intrinsic fluorescence enhancement response. Meta II decay kinetics of 10-min-preincubated bleached rhodopsin with HTS enhancer and disrupter compounds *versus* DMSO control, over a 150-s period of intrinsic fluorescence detection. *C* and *F*, a bar graph displaying the Meta II decay rates of rhodopsin calculated from the slopes of curves for the 150-s period after light exposure. Columns and error bars are means ± SD from three biological repeats. Effects of hit compounds #1, 3, 6, 7, 8, and 9 relative to DMSO control demonstrated statistically significant signal reduction (*p* < 0.0001). Statistical significance was calculated with the Student’s *t* test. DMSO, dimethyl sulfoxide; HTS, high-throughput screening; Meta, metarhodopsin.

To assess whether the identified compounds affected the transition from ground state to the metarhodopsin (Meta) II photointermediate state, UV-visible spectra of photoactivated isorhodopsin were recorded in the presence of the active compounds, and we maintained an appropriate detergent composition to mimic the dimeric state of isorhodopsin (42). After 10-s illumination, the difference spectra were obtained for isorhodopsin minus photoactivated isorhodopsin. UV-absorption spectra revealed that the identified hit compounds did not affect the complete transition of ground state isorhodopsin to Meta II upon illumination; both dimerization-disrupting compounds and dimerization-enhancing compounds gave spectra similar to the dimethyl sulfoxide (DMSO)-treated (control) sample (Fig. 4, *A* and *D*). Moreover, none of the compounds affected the UV spectral properties of isorhodopsin upon illumination.

However, oligomerization of isorhodopsin could affect the chromophore release from opsin. The chromophore release can be measured sensitively by the light-induced increase in intrinsic fluorescence of the Trp^265^ residue that occurs upon release of the chromophore after illumination (43). Compounds #1, 3, and 6 to 9 significantly quenched the increase in the fluorescent signal as compared with DMSO-treated isorhodopsin (Fig. 4, *B* and *E*), suggesting that release of the chromophore could be impaired or that these compounds interact directly with the Trp^265^ residue. Notably, the initial rates of change in fluorescence clearly demonstrated significant isorhodopsin functional changes associated with the identified compounds (Fig. 4, *C* and *F*). These results suggest that either Trp^265^ interacts *via* a π–π interaction with the identified hit compounds or allosteric binding of the hit compounds affects the rates of Meta II decay (44).

### The role of rhodopsin dimerization in rod phototransduction

Disrupting or enhancing rhodopsin dimerization did not affect activation of rhodopsin to Meta II but slowed down Meta II decay *in vitro*. Phototransduction in intact rod cells occurs in disc membranes and involves activation of G proteins, a process that could be rate-limited by diffusion of photoactivated rhodopsin and/or G proteins, as well as by inactivation of photoactivated rhodopsin by GPCR kinase 1 and arrestin 1 (45, 46, 47, 48, 49, 50). Thus, it is possible that rhodopsin dimerization plays a role both in phototransduction activation and deactivation by changing the efficiency of diffusion and/or interactions of photoactivated rhodopsin with other phototransduction components. Interference with individual components of phototransduction by these active hit compounds could be informative, but they were tested originally in very diluted conditions. Thus, we opted to use well-established physiological approaches.

**Figure 5:**
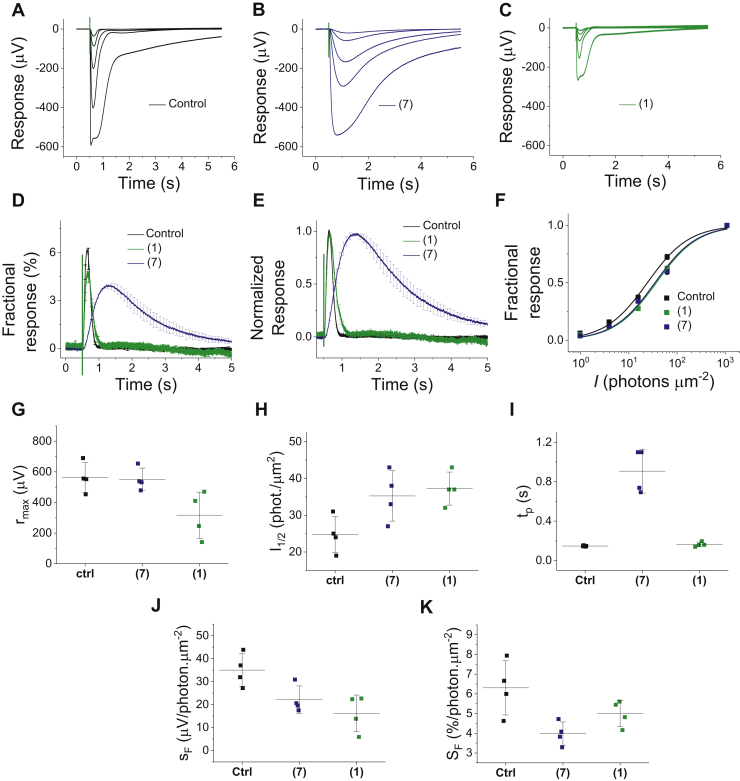
**Effects of rhodopsin dimer disrupter (#7) and promoter (#1) on rod photoreceptor light responses recorded using *ex vivo* ERG on C57 mice.***A*–*C*, representative responses to light flashes (5 ms, timing indicated by *green vertical lines*) ranging from 1 to 1100 photon μm^−2^ at 500 nm from retinas incubated in control (0.1% DMSO in Ames’ medium, *A*), (#7) (250 μM in Ames’ medium, *B*) and (#1) (250 μM in Ames’ medium, *C*). The average response (mean ± SE) to 1 photon μm^−2^ normalized with *R*_*max*_ (*D*) or peak amplitude (*E*) from retinas incubated with the control (*black*, N = 4), (#1) (green, N = 4) or (#7) (*blue*, N = 4). *F*, response amplitudes normalized with *R*_*max*_ (mean ± SE) plotted as a function of light flash intensity (*I*) from retinas incubated in the control (*black*), (#7) (*blue*) or (#1) (*green*). Smooth traces represent the best-fitting Michaelis–Menten equation with half-saturating *I*_*1/2*_ = 24 photons μm^−2^ (control), 34 photons μm^−2^ (#7), and 37 photons μm^−2^ (#1). *G*–*K*, scatter plots of rod amplitude/sensitivity and light response kinetic parameters with error bars corresponding to SD. The mean parameter values and statistical analyses of these parameter values are shown in Table 1. Absolute sensitivity (s_F_) = dim flash response amplitude divided by the flash intensity and fractional sensitivity (S_F_) = s_F_/r_max_. Light flash timing is indicated with a *vertical turquoise line* in panels *A*–*E*. DMSO, dimethyl sulfoxide; ERG, electroretinogram; I_1/2_, half-saturating intensity determined from data of individual retinas as explained above; *R*_*max*_, maximum response amplitude measured from the response to the brightest flash (1100 photon μm^−2^); t_p_, time-to-peak measured from the response to the dimmest flash (1 photon μm^−2^).

We determined the impact of compound #7 (representative disrupter of rhodopsin dimerization) and compound #1 (representative enhancer) on the sensitivity and kinetics of rod light responses, using *ex vivo* electroretinogram (ERG) analysis of isolated mouse retinas (Experimental procedures). This technique allows robust recordings from intact retinas and is well suited for quantitative investigation of mouse phototransduction (48, 49, 50, 51, 52, 53). Figure 5, *A*–*C* shows representative responses to flashes of light ranging from 1 to 1100 photons μm^−2^ (at 500 nm) from retinas that were incubated in the control medium (Ames’ medium, 0.1% DMSO) or in Ames’ medium containing 250 μM of either disrupter #7 or enhancer #1. It is evident that disrupter hit compound #7 caused a marked deceleration of light responses, whereas enhancer hit compound #1 did not substantially affect the kinetics of responses but did cause a reduction in response amplitudes.

By comparing the dim flash response amplitudes normalized by the light flash intensity, we found that the absolute phototransduction sensitivity of rods (*s*_*F*_) was reduced by both compound #7 and compound #1 (Fig. 5*J* and Table 1). After normalizing with the maximal response amplitude (*R*_*max*_), the fractional sensitivity *S*_*F*_ and the light flash intensity required to elicit 50% of the *R*_*max*_ (*I*_*1/2*_) still showed a statistically significant reduction in sensitivity, although the differences were subtle (Fig. 5, *D*, *F*, *H* and *K* and Table 1). Conversely, light response activation and recovery kinetics slowed down significantly in retinas that were incubated with the disrupter compound #7, whereas the enhancer compound #1 did not have a noticeable effect on response kinetics (Fig. 5*E*; t_p_ values in Fig. 5*I* and Table 1). These results are consistent with the idea that rhodopsin dimerization plays an important role both in phototransduction activation and deactivation. In contrast, enhancing rhodopsin dimerization did not have significant effects on response kinetics but caused a significant reduction in *R*_*max*_.

**Table 1:** Rod phototransduction parameters based on *ex vivo* ERG recordings

Compound	R_max_ (μV)	I_1/2_ (photons∙μm^−2^)	t_p_ (ms)	s_F_ (μV/photons∙μm^−2^)	S_F_ (%/photons∙μm^−2^)
Ctrl	560 ± 50	25 ± 2	147 ± 3	28 ± 8	6.3 ± 0.7
F5897-0190	550 ± 40	35 ± 3^a^	910 ± 100^a^	18 ± 6^a^	4.0 ± 0.3^a^
F2502-0030	320 ± 80^a^	37 ± 2^a^	163 ± 12	13 ± 5^a^	5.0 ± 0.3^a^

Abbreviations: *I*_*1/2*_, light flash intensity required for half-maximal response; s_F_, absolute sensitivity defined as the dim flash response amplitude divided by flash intensity (*I*); *S*_*F*_, *s*_*F*_/*r*_*max*_.; *t*_*p*_, time-to-peak of the dim flash (*I* = 1 photon μm^−2^).

N = 4 retinas for each condition.

a *p* < 0.05, Student's *t* test.

**Figure 6:**
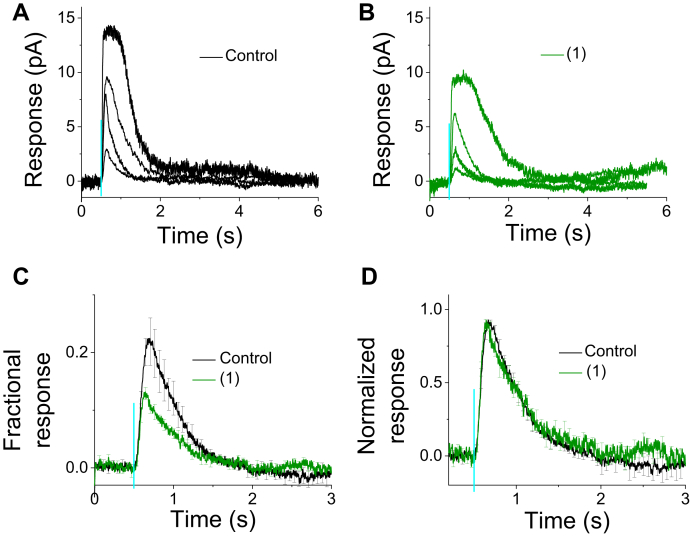
**Effect of rhodopsin dimer enhancer (#1) on rod photoreceptor light responses recorded from individual cells from C57BL/6J mouse retinas, using the suction electrode technique.***A* and *B*, representative responses to light flashes (5 ms, timing indicated by *green vertical lines*) ranging from 8 to 700 photon μm^−2^ at 500 nm from retinas incubated with control (0.1% DMSO in Ames’ medium, *A*), (#1) (250 μM in Ames’ medium, *B*). Average response (mean ± SE) to 8 photon μm^−2^ normalized with *R*_*max*_ (*C*) or peak amplitude (*D*) from retinas incubated in the control (*black*, N = 8), or (#1) (*green*, N = 10). Light flash timing is indicated with a *vertical turquoise line*. DMSO, dimethyl sulfoxide; *R*_*max*_, maximal response amplitude.

Because it is possible that reduction of *R*_*max*_ by compound #1 is due to some effect(s) on the rod photoreceptor inner segment currents that are not directly related to phototransduction (51, 52), we conducted also single-cell suction electrode recordings from the ROS from samples that were incubated either in the control medium or in the medium containing enhancer compound #1. The results from single cell recordings were similar to those obtained using *ex vivo* ERG: *R*_*max*_ and fractional sensitivity were decreased from 12 (±0.4) to 9 (±0.3) pA and from 3.0 (±0.5) to 1.8 (±0.1)% per photon μm^−2^, respectively, by rhodopsin dimerization–enhancing hit #1 (Fig. 6, *A*–*C*). Like the *ex vivo* ERG recordings, light response kinetics was not affected by compound #1 in the single-cell recordings (Fig. 6*D*). Although it was of great interest to us, we were not able to pursue single-cell experiments with compound #7 because a long incubation time with compound #7 caused damage to the photoreceptor cells.

In summary, we found that compound #7 had a significant effect on rod light response kinetics, whereas hit #1 did not significantly affect response kinetics but caused a subtle reduction of rod sensitivity and *R*_*max*_. The lack of effect of compound #1 on response kinetics is consistent with a large fraction of rhodopsin already being aggregated (3, 7, 14, 53), so the enhancer would have limited impact in intact cells.

## Discussion

In this study, we report the use of robust cell-based assays capable of identifying compounds that modulate rhodopsin dimerization (Fig. 1). Using the β-gal complementation assay as a primary screening system, we were able to identify compounds acting as rhodopsin dimerization enhancers or disrupters *in vitro*. To ensure that the signal generated is associated with rhodopsin modulation rather than activation of rhodopsin complementary units, we used the β-gal activity counter screen, and we documented that activity is not affected by the fusion proteins. This β-gal complementation assay identified 36 hits acting as potential rhodopsin modulators, including 24 disrupter molecules and 12 enhancer molecules. To determine the reliability of the screen, we used the Z′ factor, which is a widely used parameter to determine the robustness of an assay. In our screening system, the Z′ factor was 0.75, which is considered an excellent score for HTS (Fig. 2). To further validate our screening system, we randomly repeated four plates and identified ∼80% of the initial hits generated from the same plates. Furthermore, using an independent secondary BRET-based complementation assay, we reduced false positives among the initial hits, leading to nine final hit compounds comprising five disrupter molecules and four enhancer molecules. All nine compounds demonstrated dose-dependent activity in both cell-based assays, without affecting the complementary fusion proteins (Fig. 3). Among the hits identified, compound #4 unusually showed disruption in the β-gal complementation assay but enhancement in the BRET-based assay, suggesting that enhancement or disruption highly depends on the orientation and expression levels of rhodopsin in the cellular system.

All nine hits were subjected to rhodopsin photobleaching analysis with UV-visible absorption spectroscopy. None of the hits showed perturbations at the chromophore-binding site of rhodopsin, suggesting that the active compounds operate as allosteric effectors (Fig. 4, *A* and *D*). To gain insight into the allosteric binding of these compounds, a Trp fluorescence assay was carried out. All nine hits quenched the increase in fluorescence of Trp^265^, displaying a range of affinities (Fig. 4, *B* and *E*). We infer that the identified compounds affect the retention of retinal in the chromophore-binding pocket of isorhodopsin and thereby cause quenching of Trp^265^ fluorescence.

It is now well documented that rhodopsin and other GPCRs form dimers (10, 14, 29, 54, 55, 56, 57, 58, 59). Here, we used an electrophysiological approach to determine the potential role of rhodopsin dimerization in rod phototransduction, a well-defined GPCR-signaling cascade. Measurement of a highly amplified electrical signal from rods in response to quantifiable light input enables detailed investigation of the sensitivity and kinetics of rhodopsin-mediated G protein signaling in intact cells. Thus, by analyzing the impact of dimerization-disrupting or dimerization-enhancing hits from our *in vitro* assay on rod light responses, we were able to study the role of rhodopsin dimerization in its native environment. This is important as *in vitro* studies cannot recapitulate the crowded molecular environment and interactions of rhodopsin with multiple other G protein signaling components in the rod disc membranes. Our main finding was a significant deceleration of light response kinetics by disruption of rhodopsin dimerization (Fig. 5*E*). We can also dissect the effect of rhodopsin dimerization on the amplification of activation reactions by plotting dim light responses normalized with *R*_*max*_ (Fig. 5*D*). In this plot, the kinetics of the rising edge of the light response is proportional to the gain of the phototransduction activation reactions. This gain was significantly reduced by disruption of rhodopsin dimerization. Although there could be several explanations for this effect, the most probable interpretation is that rhodopsin dimerization enhances the rate of G protein activation. In contrast to the rhodopsin dimer disrupter, the rhodopsin dimerization enhancement by compound #1 did not significantly affect the gain of phototransduction activation reactions or overall kinetics of light responses (Fig. 5, *D* and *E*). The most straightforward explanation is that rhodopsins are already optimally dimerized in the rod disc membranes so that the enhancer cannot further promote the gain or speed of the rod phototransduction.

Here, we used mainly the *ex vivo* ERG technique as the electrophysiological method to record rod photoreceptor light responses from isolated WT mouse retinas. The *ex vivo* ERG technique has several advantages over single cell recordings, including high signal-to-noise ratio, objective data collection (experimenter cannot select the cells), minimal mechanical stress to photoreceptor cells, and long stable recordings. However, it has been shown that saturated rod photoreceptor ERG responses contain a component that is not directly related to the phototransduction but rather to voltage-gated channels and/or capacitive currents (52, 57). Thus, to determine if the reduced *R*_*ma*x_ elicited by compound #1 (Fig. 5*C* and Table 1) reflects reduction of the light-sensitive cyclic nucleotide–gated channel current, we performed single-cell suction electrode recordings from the ROS in retina samples that were incubated for 5 h in compound #1 (Fig. 6). These experiments also showed a reduction of *R*_*ma*x_; however, the change was significantly less than that seen with the *ex vivo* ERG experiments. On the other hand, the effects of compound #1 to dim flash response kinetics and sensitivity of the rods were identical between ERG and suction electrode experiments. However, we were not able to pursue single-cell experiments with compound #7 because we found that long incubation time with compound #7, combined with mechanical stress associated with suction electrode sample preparation, made cells more fragile. Our overall conclusion, based on *ex vivo* ERG data from compounds #1 and 7, and from single-cell recordings with compound #1, is that the intrinsic properties of dimerized rhodopsin in the rod disc membranes are important for setting the gain and kinetics of rod phototransduction.

In addition to peptides that disrupt rhodopsin dimerization (37), this study demonstrates the ability to identify small molecules that enhance or disrupt interactions between rhodopsin molecules. Thus, the identified compounds open the possibility of studying other pertinent features of GPCR function. But this work also has its limitations. First, in the case of rhodopsin and its very high density and dimer concentration in the membrane, high concentrations of the effector compounds on the order of hundreds of μM would be necessary to disrupt or enhance the dimer formation under physiological conditions. This relationship could lead to a second limitation, namely that these lead compounds would have to be highly water soluble and membrane permeable. Perhaps these problems will be of lesser importance for other GPCRs. Future efforts are required to improve on these compounds using medicinal chemistry. The richness of the core structure will allow for advancing the chemical specificity, limiting toxicity, controlling metabolism, and optimizing solubility and membrane partition. Such next-generation compounds could be valuable modifiers of GPCR signaling. A third limitation in the interpretation of our data could arise from additional effects on phototransduction of our hit compounds when used at these high concentrations. Additional approaches are needed to alleviate these concerns.

## Experimental procedures

### Mice

C57BL/6J mice were purchased from the Jackson Laboratory (Jackson Laboratory; 000664). All mice were housed at the vivarium at the University of Utah, where they were maintained on a normal mouse chow diet and a 12 h/12 h light/dark cycle. The mice arrived at the vivarium at 2 months of age and were used for experiments at 3 months of age. All animal procedures were approved by the Institutional Animal Care and Use Committee of the University of Utah (protocol #20-07015) and were conducted in accordance with the Association for Research in Vision and Ophthalmology Statement for the Use of Animals in Ophthalmic and Visual Research.

### Chemicals

The chemical library containing 50,000 compounds was purchased from Life Chemicals. 9-*cis*-retinal, β-gal activity assay substrate 4-methyl-umbelliferyl-β-d-galactopyranoside and lauryl maltose-neopentyl glycol (LMNG) were obtained from Sigma-Aldrich. The Gal-Screen β-gal reporter system for mammalian cells was purchased from Thermo Fisher Scientific. Hit compounds F2502-0030 (#1), F5103-0385 (#2), F5097-2767 (#3), F3382-0749 (#4), F1669-0696 (#5), F3215-0002 (#6), F5897-0190 (#7), F0834-0928 (#8), and F2515-3945 (#9) were obtained from Life Chemicals. BRET assay substrate pivaloyloxymethyl acetoxycoelenterazine h was obtained from Dalton Research Molecules. Rhodopsin-derived TM5 peptide (SKSKSKNESFVIYMFVVHFIIPLIVIFFSYGQL VFW-NH_2_) was custom-synthesized by EZBiolab.

### Cell line generation and HTS–β-gal complementation assay

U2OS cells were used to express a C-terminal opsin fused with EA and/or PK, as described previously (41). In short, constructs of PathHunter rhodopsin–enzyme acceptor (EA) and rhodopsin–ProLink peptide donor (PK) adherent retroparticles were generated by DiscoveRx for the ligand-induced β-gal complementation opsin dimerization assay. The U2OS cells were plated 1 day before retroviral transfection. Transduced cells were transferred to a 48-well cell culture dish containing 400 μl/well of 5 × 10^4^ cells/ml medium. For positive selection of opsin-EA–expressing cells, 300 mg/ml hygromycin B (DiscoveRx) was used and incubated for 10 days under selection at 37 °C in 5% CO_2_. Expression of the opsin-EA fusion was confirmed by immunoblotting with the mouse monoclonal B630 anti-rhodopsin antibody (molecular mass of opsin-EA, 150 kDa) and by immunostaining with the B630 anti-rhodopsin antibody, PathHunter anti-EA antibody (DiscoveRx), and the Cy3 conjugated goat anti-mouse IgG.

In this study, cultured U2OS opsin-EA and opsin-PK cells were diluted to 2 × 10^5^ cells/ml in the culture medium containing 10% fetal bovine serum (Thermo Fisher Scientific) and 1% penicillin-streptomycin 10,000 U/ml (Thermo Fisher Scientific). Cell diluent of 20 μl/well was dispensed into a white ViewPlate-384 (PerkinElmer) using the EL406 plate dispenser (BioTek). As a control, we used U2OS opsin-EA–only expressing cells as a positive control for dimerization disruption generating a minimal luminescence signal, as previously described (41). The plates were cultured overnight at 37 °C in 5% CO_2_, and the next day, under a dim red light, cells were treated with 5 μl/well of 9-*cis*-retinal (Sigma-Aldrich) at 7.5 μM final concentration. Plates were covered with aluminum foil and cultured overnight at 37 °C in 5% CO_2_. Next, cells were treated with each of the 50,000 library compounds (Life Chemicals Inc) at a final concentration of 57.6 μM, using a JANUS automated workstation (PerkinElmer), followed by incubation overnight at 37 °C, covered with aluminum foil. The next day, cells were treated with Galacton-Star chemiluminescent substrate (23 μl/well; Thermo Fisher Scientific). The plates were covered with foil and incubated at 20 °C for 2 h, followed by luminescence reading with the EnSpire multimode plate reader (PerkinElmer). Measurements were evaluated with the lowest value being defined as −50% (dimer enhancers) and the highest value as 100% (dimer disrupters) in the compound dataset. All experiments were done under red-light conditions. Identified compounds were retested in triplicates ranging from 180 μM down to 2.4 nM concentrations.

The quality control parameters, the S/B ratio, and Z′ values were calculated as S/B ratio = mean_100% control_/mean_0% control_ and Z′ = 1 – 3 × (SD_0% control_ + SD_100% control_)/|mean_100% control_ – mean_0% control_| (39). Here, the 100% control consisted of opsin-EA–expressing cells, and the 0% control had opsin-EA/PK–expressing cells. The quality control parameters demonstrated an S/B ratio greater than 123 ± 14.4 and Z′ greater than 0.75 ± 0.01.

### BRET assay

Identified hits from the β-gal complementation assay were subjected to the previously developed BRET assay, using human embryonic kidney 293 opsin–Renilla luciferase and opsin-Venus cell lines (37). Cultured cells were diluted to 2 × 10^5^ cells/ml in the medium containing 10% fetal bovine serum (Thermo Fisher Scientific) and 1% penicillin-streptomycin (10,000 U/ml; Thermo Fisher Scientific), and 20 μl/well of the cell diluent was dispensed into a white ViewPlate-384 (PerkinElmer). Cells were cultured overnight at 37 °C in 5% CO_2_, and under a dim red light, cells were treated with 9-*cis*-retinal at 7.5 μM final concentration. The next day, cells were treated with the identified hit compounds from the β-gal complementation assay at 180 μM down to 2.4 nM concentrations, and 1 μM final concentration of TM5 peptide (SKSKSKNESFVIYMFVVHFIIPLIVIFFSYGQLVFW-NH_2_; EZBiolab) serving as the control. On the following day, the culture medium was aspirated and replaced with 90 μl/well of PBS containing pivaloyloxymethyl acetoxycoelenterazine h (Dalton Research Molecules) at 600 μM concentration followed by incubation at 20 °C for 40 min (35). Dual luminescence readings at 480 and 530 nm were performed using a SpectraMax L plate reader with the BRET1 filter set (Molecular Devices).

### β-gal activation assay

Hit compounds confirmed with the BRET assay were subjected to the previously developed β-gal activation assay, using commercially available β-gal protein (Sigma-Aldrich) (41). With this assay, we tested the effect of identified hit molecules on β-gal self-association. Briefly, galactosidase protein was dissolved in 5 mM sodium phosphate buffer, pH 7.4, at 60 μM concentration; the dissolved protein was then dispensed into a black ViewPlate-384 (PerkinElmer) at 20 μl/well. Next, hit compounds ranging from 180 μM down to 2.4 nM were incubated with the protein for 2.5 h on ice. The fluorogenic substrate, 4-methylumbelliferyl-β-D-galactopyranoside (Sigma-Aldrich), was added at 20 μl/well to achieve the final concentration of 1 mM (34) and then incubated for 2 h on ice, and the enzymatic activity was monitored according to fluorescence increases at 455 nm with an EnSpire multimode plate reader (PerkinElmer).

### Rhodopsin spectroscopy

A detailed description of the isolated and prepared bovine ROS was reported previously (58). The washed ROS with isotonic and hypotonic buffers was used, in the dark under a dim red light (>670 nm) for observance, to extract rhodopsin as described previously (59, 60, 61, 62, 63, 64). Solubilized rhodopsin at 1.05 mg/ml in 20 mM 1,3-bis(tris(hydroxymethyl)methylamino) propane, pH 6.9, 100 mM NaCl, and 1 mM LMNG buffer was incubated for 5 min at 20 °C with 0.7 μl of 100 mM stock solutions of each of the nine identified hit compounds in DMSO (100 μM final concentration) and centrifuged at 1000*g* for an additional 5 min. Absorption spectra of rhodopsin (dark, and treated with the nine hit compounds and untreated) were then measured with a Cary 50 UV-visible spectrophotometer (Varian). Next, photobleaching was carried out with a 150-W fiber light delivered through a 480 to 520 nm band pass filter (Chroma Technology) for 10 s; immediately then, absorption spectra were measured again for each sample in triplicate.

### Rhodopsin Meta II decay assay

The washed ROS with isotonic and hypotonic buffers was used as described above. The solubilized rhodopsin at 1.05 mg/ml in 20 mM 1,3-bis(tris(hydroxymethyl)methylamino) propane, pH 6.9, 100 mM NaCl, and 1 mM LMNG buffer was incubated for 5 min at 20 °C with 0.7 μl of 100 mM stock solutions of the nine identified hit compounds (100 μM final concentration) in DMSO and centrifuged at 1000*g* for an additional 5 min. Next, photobleaching was carried out with a 150-W fiber light delivered through a 480 to 520 nm band pass filter (Chroma Technology) for 10 s. Samples were subjected to fluorescence measurements acquired through 150 s in triplicate. The results were analyzed with an L55 luminescence spectrophotometer (PerkinElmer) operating at excitation and emission wavelengths of 300 nm and 335 nm, respectively.

### Cell viability

The nucleic acid stain Hoechst 33342 (Sigma-Aldrich) was used as a cell-permeant nuclear counterstain, as described previously (40). Briefly, cells were washed with PBS and then incubated with 3 μM Hoechst 33342 for 15 min at 37 °C with 5% CO_2_; fluorescence visualization was confirmed with an ImageXpress Micro Confocal system (Molecular Devices).

### *Ex vivo* ERG

*Ex vivo* ERG recordings were conducted with isolated retinas of C57Bl/6J mice, as described (41, 62, 63). Mice were dark-adapted overnight and euthanized by CO_2_ asphyxiation, and whole retinas were dissected under dim red light. Before recordings, retinas were incubated for 5 h in Ames’ medium (Sigma-Aldrich) inside a light-tight box that was supplied with humidified 95% O_2_/5% CO_2_. For control retinas, 0.1% DMSO was added to the incubation medium, and for others, the incubation medium contained either 250 μM F2502-0030 (compound #1) or F5897-0190 (compound #7). These compounds were first dissolved in DMSO at 250 mM concentration, and 0.1% of this stock solution was added to the incubation medium (Ames’ medium). Stock solution was stored at −20 °C and used within a week. After incubation, retinas were placed on the *ex vivo* ERG specimen holder and perfused with Ames’ medium (saturated with 95% O_2_/5% CO_2_) at 1.5 ml/min at 35 °C. Ames’ medium was supplemented with 100 μM BaCl_2_ and 40 μM DL-AP4 (Tocris Bioscience) to isolate the photoreceptor component of the ERG signal. Responses were recorded to 5 ms flashes of light (630 nm) ranging from 276 to 308,534 photons μm^−2^, corresponding to 1 to 1100 photons μm^−2^ at 500 nm (64). Sensitivity of rod photoreceptors was determined in three ways: (1) *s*_*F*_ = peak amplitude of dim flash response divided by flash energy (1 photon μm^−2^); (2) *S*_*F*_ = *s*_*F*_/*R*_*max*_, where *R*_*max*_ is the maximal response amplitude measured at the plateau from a saturated rod response; (3) *I*_*1/2*_: flash energy required to elicit 50% of the *R*_*max*_ determined by fitting a Naka-Rushton function to flash energy-amplitude data.

### Suction electrode recording

Suction electrode recordings from individual rod photoreceptors of dark-adapted C57BL/6J mice were conducted as described previously (65). Before recordings, isolated retinas were incubated in the control medium (Ames’ medium + 0.1% DMSO) or in the hit compound–containing medium (Ames’ medium +250 μM F2502-0030 (#1)) as described above for *ex vivo* ERG experiments. After incubation, one half of the retina was chopped with a razor blade and transferred into a chamber where it was perfused at 1 ml/min with Ames’ medium saturated with 95% O_2_/5% CO_2_ at 35 °C. Recordings were made from a single ROS, using borosilicate glass electrodes with ∼1.5-μm opening (resistance ∼2 MΩ at 35 °C). The pipette solution was Hepes buffered Ames’ medium composed of 0.88 g Ames’ powder (A1420, Sigma-Aldrich), 0.357 g Hepes (Sigma-Aldrich), and 58.44 mg NaCl in 10 ml distilled and vacuum-filtered water. The pH was adjusted to 7.4 with NaOH. Light responses were recorded to flashes of light ranging from 8 to 700 photons μm^−2^ at 500 nm. For analysis, data were low-pass filtered using an 8-pole Bessel filter with cut-off frequency at 30 Hz.

### Western blot analysis

U2OS opsin-EA/PK stable cells and U2OS opsin-EA cells were lysed and immunoblotted as previously described (41). Briefly, cells were pelleted and resuspended in 30 ml PBS supplemented with 0.5 μl benzonase (MilliporeSigma) for 5 min. Next, cells were sonicated at room temperature in a water bath for 5 min at a low power and then centrifuged at 16,000*g* for 15 min at 4 °C. Total protein from cell lysates was separated by SDS-PAGE followed by transfer onto a polyvinylidene difluoride membrane. The polyvinylidene difluoride membrane was blocked with 5% unsaturated milk and incubated with mouse monoclonal B630 anti-rhodopsin monoclonal antibody (stock solution of 2 mg/ml) at a dilution of 1: 1000. Immunoblots were developed with a Novex BCIP/NBT Detection Kit (Thermo Fisher Scientific).

## Data availability

All data are contained within the article. Data from intermediate screening are available upon request to the corresponding author (kpalczew@uci.edu).

## Conflict of interest

K. P. is a Chief Scientific Officer of Polgenix, Inc and the Donald Bren Professor and the Irving H. Leopold Chair of Ophthalmology at UCI. F. V. is a recipient of a Research to Prevent Blindness/Dr H. James and Carole Free Career Development Award. T. G. and A. K. declare that they have no conflicts of interest with the contents of this article.
